# pyGeno: A Python package for precision medicine and proteogenomics

**DOI:** 10.12688/f1000research.8251.2

**Published:** 2016-05-10

**Authors:** Tariq Daouda, Claude Perreault, Sébastien Lemieux

**Affiliations:** 1 Institute for Research in Immunology and Cancer, Université de Montréal, Montreal, Canada; 2 Department of Biochemistry, Faculty of Medicine, Université de Montréal, Montreal, Canada; 3 Division of Hematology, Hôpital Maisonneuve-Rosemont, Montreal, Canada; 4 Department of Medicine, Faculty of Medicine, Université de Montréal, Montreal, Canada; 5 Department of Computer Science and Operations Research, Faculty of Arts and Sciences, Université de Montréal, Montreal, Canada

**Keywords:** Bioinformatics, python, database, precision medicine, genomics, proteomics, proteogenomics, genetic polymorphisms

## Abstract

pyGeno is a Python package mainly intended for precision medicine applications that revolve around genomics and proteomics. It integrates reference sequences and annotations from Ensembl, genomic polymorphisms from the dbSNP database and data from next-gen sequencing into an easy to use, memory-efficient and fast framework, therefore allowing the user to easily explore subject-specific genomes and proteomes. Compared to a standalone

program, pyGeno gives the user access to the complete expressivity of Python, a general programming language. Its range of application therefore encompasses both short scripts and large scale genome-wide studies.

## Introduction

High-throughput systems biology and precision medicine applications require the integration of data from many different sources. For instance, a significant part of precision medicine research revolves around the identification of relevant single nucleotide polymorphisms (SNPs) and insertions/deletions (INDELS) and the study of their context ^1^. Furthermore recent studies in proteogenomics show that replacing traditional reference databases such as Uniprot ^2^ by customized databases that integrate the subject’s genomic polymorphisms, can significantly improve the identification of peptides or proteins using mass spectrometry ^3– 6^. These applications usually require the integration of reference sequences, reference genome annotations, specific SNPs and INDELs along with an external SNP database such as dbSNP ^7^ for validation. The sheer amount of data generated by theses studies rules out most spreadsheet analyses and requires tools that are both fast and memory efficient. Furthermore, these studies often require the collaboration of people with different sets of skills. Thus, it was important to us to develop a tool that is powerful enough to be integrated in complex high-throughput pipelines, while still being understandable by users with limited technical abilities. In contrast to other projects such as BioPython ^8^ and PyCogent ^9^ whose objective is to provide a general set of tools for bioinformatics, the primarily ambition behind pyGeno is to provide the community with a powerful genome and proteome exploration tool that can be easily integrated into scripts. The current version integrates gene set annotations and reference sequences from Ensembl ^10^ along with polymorphisms (both SNPs and INDELs) derived from dbSNP ^7^, and experimentally detected patient-specific polymorphisms.

To our knowledge pyGeno is the only available tool that provides this kind of integration in an easy-to-use and programming-friendly environment. Furthermore, more advanced users can rely on object-oriented inheritance to extend the functionalities of pyGeno to implement support for polymorphisms from other sources. pyGeno has been used with human and mouse genomes and should readily work with any diploid organism whose annotations are made available by Ensembl.

## Methods

### Design and implementation

**Figure 1.**
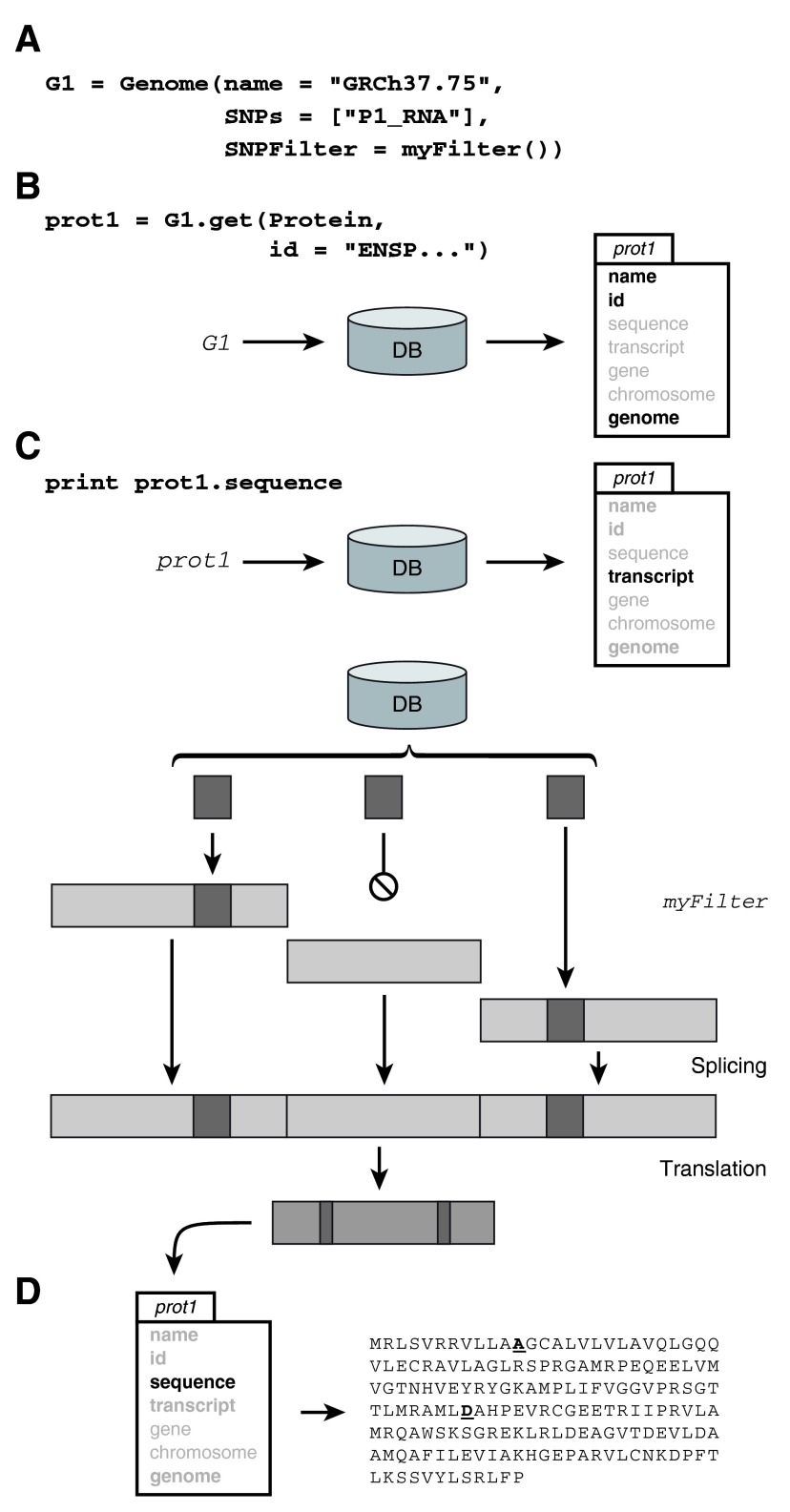
Extracting the subject-specific sequence of a protein. ( **A**) Here we instantiate a personalized genome G1 by providing the Genome constructor with the name of a reference genome, a set of polymorphisms and a user defined SNP filter (for example a quality filter). ( **B**) We then ask the get function of G1 to return a protein by id. The result is an object where only the fields in bold are fully loaded, other fields will be automatically loaded when and if accessed. ( **C**) Asking for the currently unloaded sequence of the protein triggers the following sequence of events. The transcript, as well as the exons that encode for it, and any polymorphisms in their regions are loaded. The polymorphisms are filtered according to the filter provided to the genome constructor (for example, according to sequencing quality) and inserted at their corresponding locations. The exons are then assembled into the transcript sequence and the sequence is translated. ( **D**) The sequence as well as the transcript are now fully loaded and the sequence of the precision protein is printed.

pyGeno is written in Python, a language that enjoys a large set of well established and mature scientific libraries that are used in research fields such as physics, mathematics and bioinformatics ^8, 11– 13^. pyGeno gives users access to the full expressivity of Python to explore reference and patient-specific genomes and proteomes, by manipulating familiar objects such as genomes, chromosomes, genes, transcripts, proteins and exons. In order to make pyGeno as easy to use and learn as possible, we have created an interface where only one function, *get()*, can be used for almost any query. An example of usage can be seen in Figure 1. An integrated documentation is also available through the *help()* function.

The current version of pyGeno does not require any access to remote REST APIs. This results in more robust and faster processing since the application is not affected by connection speed or sudden changes to the server API. On the other hand it also implies that extra care must be taken regarding the optimization of the application.

Memory efficiency and speed are mainly achieved through the use of a custom lazy object-oriented database system that we have specifically written for pyGeno ( https://github.com/tariqdaouda/rabaDB). When an object is loaded through the *get()* function, only a minimal version of it is served. The object fully develops only once the user accesses a field that is not present in the minimal version ( Figure 1). The transformation is entirely transparent and does not require more memory than necessary to store the fully developed object. This is especially important, since most of the time users are only interested in specific regions of the genome, and do not require that the full genome be loaded into memory. Every loaded object is also a singleton, if the user asks for a previously loaded object, pyGeno will serve the object in memory.

Furthermore, this database system is built on top of SQLite version 3 ( http://www.sqlite.org/), a serverless relational database. Because SQLite3 uses single files to store data, pyGeno’s database can be easily backed up and shared by a simple copy/paste. Moreover, the files can be directly read, modified and analyzed through any SQLite3 client.

As with any other database system, indexes play a crucial role in determining the general performance. Within pyGeno’s database, several reference genomes along with patient-specific data and versions of dbSNP can coexist. Therefore building indexes for all the stored information would result in unnecessarily large databases. We therefore have taken the approach of giving the end user full control over indexation through the *ensureGlobalIndex()* and *dropGlobalIndex()* functions. Users can, for example, decide to index the field *’id’* of transcripts by using *Transcript.ensureGlobalIndex(’id’)* and dramatically improve queries based on transcript ids.

pyGeno’s database is populated through imports of datawraps using *importSNPs* and *importGenome* functions. Datawraps are compressed archives that can be shared among co-workers, and are designed to solve the version and update problems. A datawrap contains at least one file named manifest.ini that contains basic information about the package such as a description, a version and a maintainer, as well a list of files from which data must be extracted. It is possible to either compress these files within the archive, or to specify URLs from which the files can be downloaded.

In an effort to make pyGeno as easy to install as possible we have made it as dependency-free as possible. This approach has motivated our choice for SQLite3, since it is natively supported by Python 2.5 and above, and it also lead us to develop many tools that were subsequently integrated into pyGeno. Among theses tools are various functions for translating sequences, parsers for GTF/GFF, VCF, FASTA, FASTQ and CSV files, a progress bar, and an efficient way of annotating the genome called segment trees.

## Personalized genomes

One of the biggest strengths of pyGeno is to allow the user to define personalized genomes. These genomes are built by combining a reference genome with sets of polymorphisms and a filtering function that returns the alleles to be inserted at the appropriate locus ( Figure 1). Personalized genomes are a powerful tool that can go beyond the definition of patient-specific genomes. For instance, we recently used this tool to combine the results of both RNA- and DNA-seq data and create more robust personalized genomes that were used to identify protein-derived peptides by mass spectrometry ^3^. Furthermore because pyGeno loads the necessary parts of a given reference genome only once, a pyGeno application can handle several personalized genomes without significantly increasing its memory consumption.

### Operation

pyGeno’s only requirement is Python2 and we highly recommend version 2.7.6 or later. pyGeno can be easily installed using the *pip* package manager ( https://pip.pypa.io/) by typing *pip install pyGeno* into command line interface. Alternatively the latest developments can be obtained from the github repository. Once pyGeno’s installation has been completed, the first action that users must perform is the importation of a reference genome datawrap. In order to simplify the process pyGeno comes with several datawraps that can be directly listed and installed using its *bootstrap* module. If the desired reference genome is not among the ones provided, users also have the possibility to create their own from scratch by following the steps described in the documentation. After the first reference genome importation, pyGeno is fully functional and users can further expand its database by importing other reference genomes or SNP sets.

## Summary

We have developed pyGeno because, in an age where both precision medicine and DNA/RNA sequencing are becoming more and more important, we needed a tool that would allow us to easily work on personalized genomes that include subject-specific genomic features. Nowadays research teams are increasingly multidisciplinary and are composed of people with very different backgrounds. Since we wanted pyGeno to serve as a common language between users, we therefore took great care in making pyGeno easy to install, easy to use and optimized it so it can run on computers with limited resources (eg. laptops). The fact that pyGeno has been downloaded more than 12,000 times over its first year of existence suggests that there is indeed a need for powerful user-friendly precision medicine tools. With pyGeno we have taken a rather unusual approach to user-friendliness. Instead of writing a program with a graphical user interface (GUI), we have decided to create a Python module that fully integrates within the Python environment. This ensures that users can leverage the full expressiveness of Python as well as the functionalities of other python modules such as SciPy and numpy ^11^, pandas ( http://pandas.pydata.org/) and matplotlib ^13^, to meet their specific needs. Furthermore, it led us to think of the functions and objects the user manipulates as pyGeno’s interface and we strived to make it as simple and easy to learn as possible.

In the past few years great technologies have been developed. Scripting languages such as Python and JavaScript have taken programming to a whole new level of simplicity, and are now fast enough to serve as foundations to large-scale projects. Freely available libraries such as D3.js ( http://d3js.org/) allow for the creation of stunning data representations, that once coupled with tools such as pyGeno, could be used to create powerful interactive representations of biological data. The NoSQL movement has produced several new database systems from which developers can choose, offering them the opportunity to store sheer amounts of data with a flexibility that was not present only a few years ago. These technologies and many others are only waiting to be put together into ground breaking tools for the treatment of biological data. In life saving research areas, we believe that great tools that dramatically improve workflow efficiency are not a luxury but a necessity.

## Software availability

1. pyGeno is available from the Python Package Index (PyPI; https://pypi.python.org) via: pip install pyGeno.
2. Latest source code: https://github.com/tariqdaouda/pyGeno.
3. Documentation: http://pyGeno.iric.ca
4. Link to archived source code as at time of publication: https://zenodo.org/record/50587#.VyIP0UErJB0 (doi: 10.5281/zenodo.50587)
5. License: Apache License Version 2.0
