# Supplementary material for: pyGeno: A Python package for precision medicine and proteogenomics
Source: F1000Res. 2016 May 10;5:381. Originally published 2016 Mar 21. [Version 2] doi: 10.12688/f1000research.8251.2 (PMC5022704; doi:10.12688/f1000research.8251.2)
Supplement: Peer review history

# Peer review: Referee response for version 2

**DOI:** 10.5256/f1000research.9391.r15427

**Published:** 2016-09-13

**Authors:** Christian Cole

**Affiliations:** 1 Division of Computational Biology, School of Life Sciences, University of Dundee, Dundee, UK

Daouda *et al.* propose a new tool, pyGeno, for the interrogation of proteomics data in the context of genomic sequence variants.

 General Comment:

  

- Several times the authors make reference to ‘precision medicine’ without clarifying what is meant by the term. If the authors have a specific workflow or use case which befits ‘precision medicine’ they need to make it clearer. I note that the authors do not include a ‘Use Cases’ section as suggested by in the ‘Instructions to Authors’. Their previous paper (Granados *et al*, 2014) would be  a great example of how to use pyGeno on real data.The examples provided in the paper are either too vague or too simplistic.
- Too often new tools come out which try to reinvent the wheel for a small incremental improvement. Here, the authors need to be acknowledged for using well-established systems like Ensembl, SQLite and reading in existing data types (e.g. GFF, VCF, fasta).
- The paper is too short on specifics and somewhat unstructured. The Methods section is fine although would benefit from an overview- with a Figure and/ or text- as the overall structure of pyGeno is unclear. The Personalized genomes section should be expanded as a ‘Use Cases’ section. The ‘Operation’ section should be part of the Methods.
- The online instructions are quite clear for installation, however I gave up due to the incredibly slow progress: >20 hours remaining of a full genome ‘datawrap’. Thus, unfortunately, I was not able to test the software myself. Given that pyGeno has been downloaded many times, this might an issue local to me.
- Overall I do feel pyGeno is a valuable contribution to the community, however the paper needs some improvement to highlight the tool’s usefulness better.

 

 Specific Comments:

  

- In the third paragraph of the Methods, the authors state that pyGeno is not dependent on any ‘remote REST APIs’. If this is the case how does the pyGeno interact with Ensembl and keep in sync with the regular updates (every 6 months)? The focus on ‘robust and faster processing’ is understandable, but version drift from official sources can be a serious problem. Is version maintenance something end users can do or are they dependent on the authors keeping the versions up-to-date?
- In Figure 1, a simple example is provided showing a protein sequence with what appears to be two non-synonymous variants highlighted in the protein sequence. How does pyGeno cope with summarising/ visualising; 1) mutually exclusive variants at a single amino acid (e.g. two non-synonymous variants at different positions of the codon ), or 2) more complex variants like splicing-affecting changes and loss/gain of STOP codons? Similarly does pyGeno accept phased haplotypes thereby allowing inspection of both protein products from each of the individual’s alleles?
- What are the hardware requirements for running pyGeno and associated analyses? Is a well-specified workstation with several GB of RAM, fast cpu and terabytes of diskspace required or can it be run on a laptop?
- The final paragraph does not contribute anything to the paper, I suggest the authors remove it and end the article with something more succinct and pertinent.

I have read this submission. I believe that I have an appropriate level of expertise to confirm that it is of an acceptable scientific standard, however I have significant reservations, as outlined above.

## References

[1] GranadosDPSriranganadaneDDaoudaTZiegerALaumontCMCaron-LizotteOBoucherGHardyMPGendronPCôtéCLemieuxSThibaultPPerreaultC: Impact of genomic polymorphisms on the repertoire of human MHC class I-associated peptides. *Nat Commun*.2014;5: 10.1038/ncomms4600 3600 10.1038/ncomms4600 24714562PMC3996541

# Peer review: Referee response for version 2

**DOI:** 10.5256/f1000research.9391.r14063

**Published:** 2016-05-31

**Authors:** Hilary Coller

**Affiliations:** 1 Department of Molecular, Cell and Developmental Biology, University of California Los Angeles, Los Angeles, CA, USA

This is manuscript describes the development of a valuable new Python package that provides the user with an environment in which they can explore multiple different large datasets related to a single gene. The software maps genes back to Ensemble, provides polymorphisms from dbSN, and provides information on experimentally detected patient-specific polymorphisms. The software can also handle DNA Sequencing data, RNA-Seq data and proteomics data for the same individual. The authors have designed the software so that it manages these datasets efficiently, thus providing the user with seamless and rapid access to the information desired.  The use of “datawraps” so that co-authors can share information will likely also be valuable for users. The software has been downloaded over 12,000 times in the first year, demonstrating its utility. There is a very helpful figure, Figure 1, that gives an overview of the process. The authors might consider adding a section of the manuscript in which they walk the reader through the analysis of an actual gene in an actual genome to give the reader a sense of the findings.

I have read this submission. I believe that I have an appropriate level of expertise to confirm that it is of an acceptable scientific standard.

# Peer review: Referee response for version 2

**DOI:** 10.5256/f1000research.9391.r13874

**Published:** 2016-05-31

**Authors:** Lynn Fink

**Affiliations:** 1 Diamantina Institute, University of Queensland, Brisbane, Australia

pyGeno is a Python package that allows a user to simply query either a standard reference genome or a custom genome for information such as sequences, SNPs, and related RNA and protein data. I agree that this package fills a need for genome research, appears to be very straightforward to use, and I would like to use it myself.

 However, when I tested it I was unable to get it to run; contrary to the authors claims that it is easy to install with minimal dependencies I couldn't make it work. (I am not a Python expert, but I have installed much more complicated packages successfully.) With Python virtual environments, I wonder if there is a need to be quite so minimal. Would it be easier to rely on standard packages to ensure a universal, smooth experience?

I have read this submission. I believe that I have an appropriate level of expertise to confirm that it is of an acceptable scientific standard, however I have significant reservations, as outlined above.

# Response

**Published:** 2016-06-03

**Authors:** Tariq Daouda

**Affiliations:** University of Montreal, Canada

Thank you for taking the time to review our work.

 In light of your review we have retested the installation, genome importation and polymorphism insertion on Linux, MacOS and Windows and it seems to work on every platform. We are fully committed to make the experience for the end user as smooth and easy as possible, if you could give us more details about the problems you encountered by filling a GitHub issue, we would be very happy to address them.
